# In vitro cytotoxic activity of medicinal plants from Nigeria ethnomedicine on Rhabdomyosarcoma cancer cell line and HPLC analysis of active extracts

**DOI:** 10.1186/s12906-017-2005-8

**Published:** 2017-11-22

**Authors:** Omonike O. Ogbole, Peter A. Segun, Adekunle J. Adeniji

**Affiliations:** 1 Department of Pharmacognosy, Faculty of Pharmacy, University of Ibadan, Ibadan, Nigeria; 2 Department of Pharmacognosy, Faculty of Pharmacy, Olabisi Onabanjo University, Sagamu Campus, Ago-Iwoye, Nigeria; 3 Department of Virology, College of Medicine, University of Ibadan, Ibadan, Nigeria

**Keywords:** Nigeria, Ethnomedicine, Cancer, MTT assay, Brine shrimp lethality assay, *Macaranga Barteri*, *Calliandra portoricensis*

## Abstract

**Background:**

Cancer is a leading cause of death world-wide, with approximately 17.5 million new cases and 8.7 million cancer related deaths in 2015. The problems of poor selectivity and severe side effects of the available anticancer drugs, have demanded the need for the development of safer and more effective chemotherapeutic agents. The present study was aimed at determining the cytotoxicities of 31 medicinal plants extracts, used in Nigerian ethnomedicine for the treatment of cancer.

**Methods:**

The plant extracts were screened for cytotoxicity, using the brine shrimp lethality assay (BSLA) and MTT cytotoxicity assay. Rhabdomyosarcoma (RD) cell line, normal Vero cell line and the normal prostate (PNT2) cell line were used for the MTT assay, while *Artemia salina* nauplii was used for the BSLA. The phytochemical composition of the active plant extracts was determined by high performance liquid chromatography (HPLC) analysis.

**Results:**

The extract of *Eluesine indica* (L.) Gaertn (Poaceae), with a LC_50_ value of 76.3 μg/mL, had the highest cytotoxicity on the brine shrimp larvae compared to cyclophosphamide (LC_50_ = 101.3 μg/mL). Two plants extracts, *Macaranga barteri* Mull. Arg. (Euphorbiaceae) and *Calliandra portoricensis* (Jacq.) Benth (Leguminosae) exhibited significant cytotoxic activity against the RD cell line and had comparable lethal activity on the brine shrimps. Further cytotoxic investigation showed that the dichloromethane fraction of *Macaranga barteri* (DMB) and the ethyl acetate fraction of *Calliandra portoricensis* (ECP), exhibited approximately 6-fold and 4-fold activity, respectively, compared to cyclophosphamide on RD cell line. Determination of selective index (SI) using Vero and PNT2 cell line indicated that DMB and ECP displayed a high degree of selectivity against the cancer cell under investigation. HPLC analysis showed that 3,5dicaffeoylquinic acid, acteoside, kampferol-7-*O*-glucoside and bastadin 11 were the major components of DMB while the major components of ECP were neurolenin B, nigrosporolide and trans-geranic acid.

**Conclusion:**

The results demonstrate the cytotoxicity of *Macaranga barteri* and *Calliandra portoricensis* extracts*,* which are used in Nigerian folklore for cancer treatment.

## Background

Cancer remains a leading cause of death worldwide, with approximately 17.5 million new cases and 8.7 million cancer related deaths in 2015 [1]. With approximately 20% of the population of Africa and slightly more than half the population of West Africa, Nigeria contributed 11% to the estimated 681,000 new cases of cancer that occurred in Africa in 2008 [2]. In Nigeria, the common cancer types include cancer of the breast, cervix, prostate, colorectal, liver cancer and Non Hodgkin Lymphoma [3]. Rhabdomyosarcoma (RD) is the commonest soft tissue sarcoma in children and adolescents below 20 years. In the US, the incidence of RD is approximately five cases per million children/adolescents per year, and, in >50% of cases, RD occurs during the first decade of life [4]. In Nigeria, rhabdomyosarcoma occurs frequently in the paediatric population, and it is a common cause of mortality in this age group. The most prevalent variant is the embryonal rhabdomyosarcoma and the commonest anatomical sites are the head and neck regions [5]. Several chemotherapeutic agents are being used for the management of cancer but the problem of selective toxicity and severe side effects still exist. Hence, there is an urgent need to discover new anticancer drug leads.

Natural products have been used since ancient times for the treatment of many diseases. Before the 20th century, 80% of all medicines used to treat human and animal illness were obtained from the leaves, barks and roots of medicinal plants. During that period, crude botanicals were percolated in readily available fluids like alcohol and the doctor will prescribe tablespoons of the fluid extract to be taken for a period. It is noteworthy to mention that about 70% of the drugs used today are models of natural products [6]. Between 1981 and 2010, approximately 700 natural products or natural product derived New Chemical Entities (NCEs) were approved [7]. Biodiversity and traditional medical knowledge had provided useful lead compounds for cancer chemotherapy, as exemplified in the discovery of the vinca alkaloids (vincristine and vinblastine), taxols (paclitaxel and docetaxel), camptothecin and etoposide [8, 9].

According to the World Health Organization (WHO), 80% of people living in developing nations, which includes Nigeria, depend on herbal medicine to meet their health care needs [10]. Nigeria is a country blessed with a vast flora of medicinal importance and the therapeutic potency of such plants have been demonstrated by several researchers [11, 12]. In Nigeria, many people especially those residing in the rural areas, solely depend on the traditional medical practitioners (TMPs) for the diagnosis and treatment of many diseases including cancer. Interestingly, several studies have documented the indigenous knowledge of the TMPs in the treatment of various cancers across the geopolitical zones of Nigeria [13–15]. Although, the cytotoxicity of some of these plants have been investigated [16, 17], there is yet to be a plant from Nigerian ethnomedicine that have produced a lead in anticancer drug discovery.

In this framework, data on plants used in the treatment of cancer by the TMPs in the Ijebu region of southwestern Nigeria were collected, by personal contact with the TMPs during an ethnobotanical survey conducted between May – September 2015. From the 90 plants that were recorded during the survey, bibliographical survey permitted to retain 31 indigenous plants for which cytotoxic activity had never been evaluated or only partially studied. The present study investigates the in vitro cytotoxicity of the methanol extract of the medicinal plants and the active fractions against *Artemia salina* nauplii and RD, Vero and PNT2 cell line.

## Methods

### Plant material

The selected plants were collected between September 2015 and May 2016 in their natural habitat (Table 1). The plants were identified and authenticated by comparison with appropriate voucher specimens at Department of Pharmacognosy, Herbarium, University of Ibadan (DPHUI) by Mr. P. Agwu and the Forest Herbarium Ibadan, Forestry Research Institute of Nigeria (FRIN), by Mr. T.K. Odewo.

**Table 1:** Plant species analysed for cytotoxicity

S/N	Plant	Family	Local name	Part used	Voucher number
1	*Acanthospermum hispidum* D.C.	Asteraceae	Dagunro gogoro	Aerial part	FHI 110050
2	*Alchornea laxiflora (Benth.) Pax & K.Hoffm.*	Euphorbiaceae	Pepe	leaf	FHI 110155
3	*Boerhavia diffusa* L.	Nyctaginaceae	Etipase-erinla	Root	FHI 109603
4	*Calliandra portoricensis* (Jacq.) Benth	Leguminosae	Tude	Root	FHI 109672
5	*Clausena anisata* (Willd.) Hook.f. ex Benth.	Rutaceae	Atabari obuko	Leaves	FHI 99457
6	*Croton gratissimus* Burch.	Euphorbiaceae	Ajekobale	Leaves	FHI 109041
7	*Eleusine indica* (L.) Gaertn	Poaceae	Gbegi	Whole plant	FHI 92140
8	*Entandrophragma utile* (Dawe & Sprague) Sprague	Meliaceae	Jebo	Stem bark	FHI 86848
9	*Ficus sur* Forssk.	Moraceae	Opoto	leaves	FHI 109329
10	*Heliotropium indicum* L.	Boraginaceae	Apari igun	Aerial part	FHI 110156
11	*Holarrhena floribunda*	Apocynaceae	Dagba	leaves	FHI 110053
12	*Hoslundia opposita* Vahl	Lamiaceae	Efirin-odan	Leaves	DPHUI 0341
13	*Ipomoea asarifolia* (Desr.) Roem. & Schult.	Convolvulaceae	Gboro ayaba	leaves	FHI 110052
14	*Lagenaria breviflora* (Benth.) Roberty	Cucurbitaceae	Tagiri	Seed	FHI 109040
15	*Lecaniodiscus cupanioides* Planch. Ex Benth.	Sapindaceae	Arika	leaves	FHI 110081
16	*Lippia multiflora* Moldenke	Verbenaceae	Eforomoba	leaves	DPHUI 0412
17	*Macaranga barteri* Mull. Arg.	Euphorbiaceae	Agbasa	Leaves	FHI 107230
18	*Mimosa pudica* L.	Mimosaseae	Patanmo	Leaves	FHI 100332
19	*Mondia whitei* (Hook.f.) Skeels	*Periplocacaea*	Isirigun	Leaves	FHI 110043
20	*Nauclea diderrichii* (De Wild.) Merr.	Rubiaceae	Opepe	Stem bark	FHI 110049
21	*Parquetina nigrescens* (Afzel.) Bullock	Apocynaceae	Ogbo	leaves	FHI 110044
22	*Petiveria alliacea* L.	Phytolacaceae	Awopa	leaves	DPHUI 0095
23	*Picralima nitida* (Stapf) T. Durand & H. Durand	Apocynaceae	Abeere	Seed	FHI 108794
24	*Piper guineensis Schumach. & Thonn.*	Piperaceae	Iyere	leaves	FHI 110051
25	*Sarcocephalus latifolius* (Sm.) E.A.Bruce	Rubiaceae	Egbesi	Leaves	DPHUI 1591
26	*Secamone afzelii* (roem et Schult) K. Schum	Asclepiadaceae	Ailu	Leaves	FHI 109995
27	*Spondias mombin L.*	Anacardiaceae	Iyeye	Leaves,	FHI 63948
28	*Terminalia ivorensis* (A. Chev.)	Combretaceae	Afara-dudu	Stem bark	FHI 105432
29	*Terminalia superba* (Engl. & Diels.)	Combretaceae	Afara	Stem bark	DPHUI 0214
30	*Tetracera alnifolia* Willd.	Dilieniaceae	Opon	Leaves	FHI 107511
31	*Trichilia monadelpha* Benth.	Meliaceae		leaves	FHI 108844

### Preparation of crude extracts and fractions

Plant parts were air-dried at room temperature and milled into coarse powder. For each plant material, 300 g of material was macerated in methanol, with intermittent stirring, for 72 h at room temperature. The extracts were filtered and concentrated to dryness using a rotary evaporator. Dry extracts were stored at 4 °C until analysis. To obtain fractions of different polarity from the active extracts, 20 g of the crude extracts (*M. barteri* and *C. portoricensis*) were re-dissolved in 250 mL of distilled water and partitioned (3×) with an equal volume of *n*-hexane, dichloromethane and ethyl acetate. Fractions were concentrated under reduced pressure.

### Brine shrimp lethality assay (BSLA)

The BSLA assay is a bench top assay used for screening natural products for the presence of bioactive compounds. The experiment was carried out using the method described by Mclaughlin [18]. Brine shrimp eggs were obtained from the Department of Pharmacognosy, University of Ibadan. Briefly, *Artemia salina* cysts (brine shrimp eggs 0.1 g) were allowed to hatch in natural sea water, containing 3.8 g/L salt, obtained from Bar beach, Ikoyi, Lagos. The larvae (nauplii) were placed in sea water for 48 h at 25 °C under constant aeration and illumination to ensure survival and maturity before use. Stock solutions (10 mg/mL) of plant extracts were made and diluted serially in clean test tubes of 10 mL volume to obtain five final concentrations (1000–1 μg/mL). Ten nauplii were collected with the aid of a pipette and added to the serially diluted test solutions. Test were carried out in triplicate. The negative control consisted of ten nauplii per tube in sea water without plant extract while cyclophosphamide was used as the positive control. After the 24 h incubation at 25 °C, a magnifying lens was used to count the number of dead larvae and the percentage mortality was calculated. Larvae were considered dead only if they did not move for few seconds after pricking with sharp object during observation. The 50% lethal concentration (LC_50_ value) and the standard error mean (SEM) were calculated using a non-linear regression curve contained in the Graph pad prism statistical software.

### Determination of effect of plant extract on cell proliferation by MTT assay

#### Cell culture

Cytotoxic studies were determined in human Rhabdomyosarcoma (RD) cells (CDC, Atlanta, USA), African green monkey kidney (Vero) cell (WHO Reference Polio laboratory, UCH, Ibadan, Nigeria) and the normal human prostate (PNT2) cell line obtained from the (WHO Reference Polio laboratory, UCH, Ibadan, Nigeria). Cells were grown in Eagle’s MEM supplemented with 10% FBS, 100 units/mL of penicillin, 100 mg/mL of streptomycin, 2 mM L-glutamine, 0.07% NaHCO_3_, and 1% non-essential amino acids and vitamin solution. Cultures were maintained in a humidified atmosphere with 5% CO_2_ at 37 °C and passaged bi-weekly.

#### Cytotoxicity assay

Cell viability was examined by the ability of the cells to cleave the tetrazolium salt MTT [3-(4,5-dimethylthiazol-2-yl)-2,5- diphenyl tetrazolium bromide (Sigma, Chem, St. Louis, MO), by the mitochondrial enzyme succinate dehydrogenase following the procedure as described earlier [19]. Briefly, each extract was pre-solubilized in dimethylsulphoxide (DMSO) at 37 °C to give a stock solution (SS) of 1 mg/mL. Serial ten-fold dilutions were made from SS to give working concentrations of 1000–0.01 μg/mL, making sure that the final concentration of DMSO in the tested dilutions was not higher than 1%. Confluent monolayers of RD/Vero cells were grown in 96 well-microtitre plates for 24 h. Cells were incubated with various concentrations of the test extracts in triplicate at 37 °C in a CO_2_ environment for 72 h. The negative control was performed using growth medium alone instead of plant extract, while cyclophosphamide (CTX) was used as the positive control. In addition, the cell viability was examined microscopically for the presence or otherwise of cytopathic effect (CPE). At the expiration of the 72 h treatment period, supernatants were removed from the wells and 25 μL of the MTT solution (2 mg/mL in PBS) was added to each well. The plates were incubated for 1.5 h at 37 °C, and 125 μL of DMSO was added to each well to dissolve the formazan crystals. The plates were placed on a shaker for 15 min and the optical density was determined at 492 nm on a multi-well spectrophotometer (Multiskan, Thermo Fisher Scientific, Waltham, MA). The 50% cytotoxic concentration (CC_50_) was defined as the extract/compound concentration required for the reduction of cell viability by half. The CC_50_ value and the standard error mean (SEM) were calculated using a non-linear regression curve contained in the Graph pad prism statistical software.

#### Selectivity index (SI)

The normal African green monkey kidney epithelial cell line (Vero) and the normal human prostate (PNT2) cell line were used to measure the SI. The SI of the most active fraction was calculated as the ratio of cytotoxic effect on normal cell line to the cytotoxic effect on cancer (RD) cell line.

#### High performance liquid chromatography (HPLC) analysis

The active fractions were subjected to HPLC analysis using the Dionex HPLC System 2695 (Waters). Reversed-phase analytical HPLC experiments were performed on a thermoscientific NX 5 μM C18 column (250 × 4.6 mm). The column temperature was set at 25 °C. A variable wavelength ultraviolet–visible detector was set at 235 nm. 0.1% trifluoroacetic acid in water (solvent A) and 0.1% trifluoroacetic acid in methanol (solvent B) was used as the eluent. For the analysis, the mobile phase composed of 70% of A and 30% B at 0 min, then linear gradient to 100% of B over 40 min and held at that composition for 10 min at a flowrate of 1 mL/min. To determine that nature of the compounds present in these active fractions, the peaks generated in the chromatogram of each active fraction were compared to the compounds collection contained in the HPLC library.

## Results

### Brine shrimp lethality assay

The result obtained showed that the activity of all the plant extracts were concentration-dependent. All the plant extracts, with the exception of *Nauclea diderrichii* extract*,* had LC_50_ value below 1000 μg/mL indicating the presence of bioactive secondary metabolites in these plants. *Eleusine indica* extract (LC_50_ = 76.3 μg/mL) had the highest cytotoxicity on the brine shrimps compared to cyclophosphamide (LC_50_ = 101.3 μg/mL). The result of the BSLA is summarized in Table 2.

**Table 2:** Brine shrimp lethal activity (BSLA) and in vitro cytotoxicity of methanol extract selected plants on RD cells

S/N	Plant	BSLA, LC_50_ (μg/mL, *n* = 3)	MTT Assay, CC_50_ (μg/mL, n = 3)
1	*Acanthospermum hispidum*	183.70 ± 5.64	19.65 ± 1.23
2	*Alchornea laxiflora*	142.40 ± 4.14	> 100
3	*Boerhavia diffusa*	424.7 ± 11.64	> 100
4	*Calliandra portoricensis*	188.5 ± 5.94	0.82 ± 0.08
5	*Clausena anisata*	318.2 ± 8.12	8.83 ± 0.59
6	*Croton gratissimus*	ND	> 100
7	*Eleusine indica*	76.3 ± 2.61	11.42 ± 1.01
8	*Entandrophragma utile*	247.1 ± 5.64	51.74 ± 3.71
9	*Ficus sur*	ND	19.23 ± 3.21
10	*Heliotropium indicum*	391.30 ± 11.24	> 100
11	*Hoslundia opposite*	149.8 ± 11.61	> 100
12	*Holarrhena floribunda*	595.10 ± 15.11	> 100
13	*Ipomoea asarifolia*	484 ± 9.12	88.21 ± 5.61
14	*Lagenaria breviflora*	273.2 ± 7.98	12.17 + 0.81
15	*Lecaniodiscus cupanioides*	ND	17.23 ± 1.98
16	*Lippia multiflora*	ND	> 100
17	*Macaranga barteri*	159.13 ± 11.12	0.22 ± 0.01
18	*Mimosa pudica*	443.2 ± 7.22	2.03 ± 0.11
19	*Mondia whitei*	ND	> 100
20	*Nauclea diderrichii*	> 1000	> 100
21	*Parquetina nigrescens*	ND	43.22 ± 2.43
22	*Petiveria alliacea*	ND	> 100
23	*Picralima nitida*	260.6 ± 9.64	55.59 ± 3.01
24	*Piper guineensis*	285.50 ± 1.16	> 100
25	*Sarcocephalus latifolius*	616.2 ± 5.63	> 100
26	*Secamone afzelii*	450.5 ± 8.61	11.99 ± 2.01
27	*Spondias mombin*	ND	53.33 ± 5.21
28	*Terminalia ivorensis*	492.9 ± 10.12	13.42 ± 0.92
29	*Terminalia superba*	161.7 ± 5.35	> 100
30	*Tetracera alnifolia*	155.1 ± 1.94	> 100
31	*Trichilia monadelpha*	ND	> 100
32	Cyclophosphamide	101.3 ± 1.12	0.97 ± 0.03

*n* no. of replicates, *ND* Not determined

### MTT assay and selectivity studies

The result obtained showed that from the selected 31 plants extracts, only eleven had cytotoxicity with CC_50_ less than 30 μg/mL (Table 2). The methanol crude extract of *Macaranga barteri* leaves had the highest cytotoxicity on RD cell line, with a CC_50_ value of 0.22 μg/mL, followed by the methanol crude extract of *Calliandra portoricensis* root with a CC_50_ value of 0.82 μg/mL, compared to cyclophosphamide (CC_50_ value = 0.97 μg/mL). Other plants with significant cytotoxic effect against the RD cell line include *Mimosa pudica*, *Clausena anisata*, *Eleusine indica*, *Secamone afzelii*, *Lagenaria breviflora*, *Terminalia ivorensis*, *Lecaniodiscus cupanioides*, *Ficus sur* and *Acanthospermum hispidum* (Table 2).

*Macaranga barteri* and *Calliandra portoricensis* were selected for further studies based on their cytotoxic activities. The crude methanol extracts of both plants were partitioned into *n-*hexane, dichloromethane and ethyl acetate. The DCM fraction of *M. barteri* (DMB) with a CC_50_ value of 0.15 μg/mL and the ethyl acetate fraction of *C. portoricensis* (ECP) with a CC_50_ value of 0.25 μg/mL showed the highest activity among the fractions. The SI result revealed that DMB had a SI value of 13.7 when compared to CTX with SI of 5.4 while the ECP showed a SI value of 11.1 (Table 3).

**Table 3:** Antiproliferative activity of fractions of *M. barteri* and *C. portoricensis*

Plant	Fractions	Antiproliferative activity CC_50_ (μg/mL)			Selectivity Index	
		RD	Vero	PNT2	Vero	PNT2
*M. barteri*	hexane	25.6 ± 0.21	62.07 ± 0.98	88.23 ± 2.11	2.4	3.4
	DCM	0.15 ± 0.01	2.06 ± 0.11	2.91 ± 0.18	13.7	19.4
	EtOAc	2.98 ± 0.09	10.87 ± 0.35	17.12 ± 0.41	3.7	5.7
	aqueous	3.06 ± 0.11	13.04 ± 0.41	18.13 ± 1.03	4.3	5.9
*C. portoricensis*	hexane	2.99 ± 0.04	ND	15.11 ± 1.02	–	5.1
	EtOAc	0.25 ± 0.03	ND	2.77 ± 0.12	–	11.1
	aqueous	3.04 ± 0.19	ND	12.12 ± 0.67	–	4.0
Cyclophosphamide	CTX	0.97 ± 0.03	5.23 ± 0.21	ND	–	5.4

*ND* not determined, *EtOAc* ethyl acetate

### HPLC analysis of active extracts

**Fig. 1:**
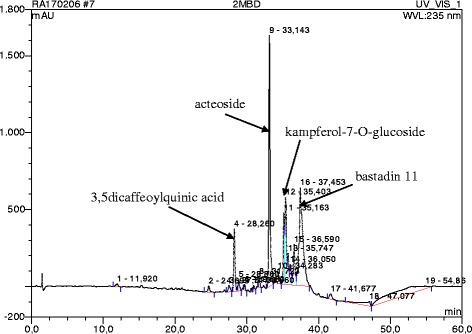
Reverse Phase-HPLC Quantitative Chromatogram of the DCM fraction of *Macaranga barteri* leaves

**Fig. 2:**
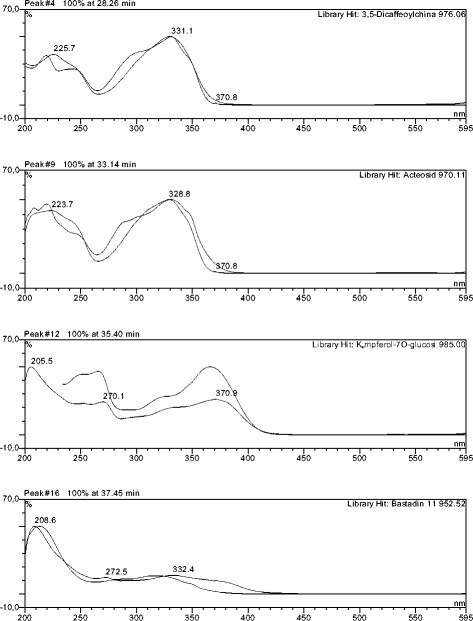
UV spectra assignment of major peaks of the DCM fraction of *Macaranga barteri* leave

**Fig. 3:**
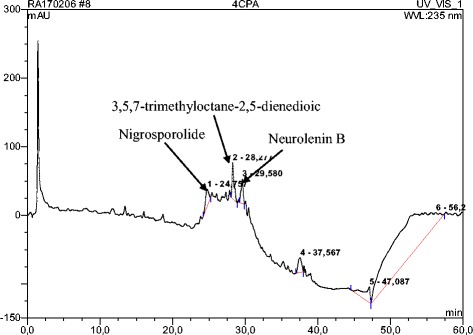
Reverse Phase-HPLC Quantitative Chromatogram of ethyl acetate fraction of the root of *Calliandra portoricensis*

**Fig. 4:**
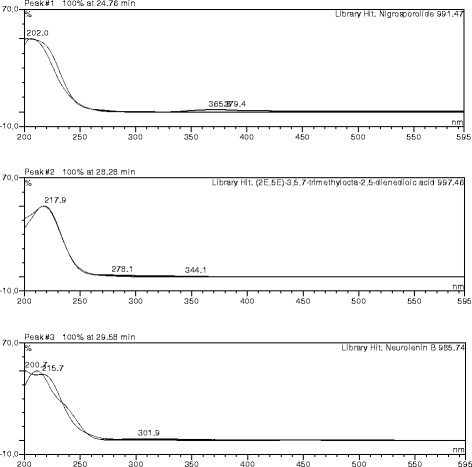
UV spectra assignment of major peaks of the EtOAc fraction of *Calliandra portoricensis* leaves

The HPLC profile of DMB shows 19 distinct peaks, with each peak demonstrating a particular chemical compound. Peak 4, 6, 12 and 16 which represents 3,5-dicaffeoylquinic acid, acteoside, kampferol-7-*O*-glucoside and bastadin 11, respectively, were the major compounds present, as observed in the chromatogram (Figs. 1 and 2). Figure 2 showed the UV spectra assignment of the major peaks of the DCM fraction. The HPLC analysis of ECP revealed the presence of neurolenin B, nigrosporolide and 3,5,7-trimethyloctane-2,5-dienedioic as the major compounds, as observed in the chromatogram (Fig. 3). Figure 4 showed UV spectra assignment of major peaks of the EtOAc fraction of ECP.

## Discussion

Thirty-one plant extracts belonging to 25 families were screened using the BSLA. *Eleusine indica* extract with LC_50_ value of 76.3 μg/mL had the highest cytotoxicity amongst the plant extracts. Although, BSLA is inadequate in determining the mechanism of action of the bioactive substances in plants nor is it specific for antitumor activity, it provides a preliminary screen that can be supported by a more specific bioassay, once the active compounds have been isolated [20]. Plants found to be toxic to brine shrimp are likely to be good candidate for anti-cancer research [21].

The MTT assay is a sensitive, quantitative and reliable colorimetric assay that measure cell viability. The assay is based on the capacity of the cellular mitochondrial dehydrogenase enzyme in living cells to reduce the yellow water-soluble substrate 3-(4,5-dimethylthiazol-2yl)-2,5-diphenyl tetrazolium bromide (MTT) into a dark blue/purple formazan product which is insoluble in water. The amount of formazan produced is directly proportional to the cell number in a range of cells lines [22, 23]. According to the American National Cancer Institute (NCI), the criteria of cytotoxicity for crude extracts is an CC_50_ < 30 μg/mL after an exposure time of 72 h in a preliminary assay [24]. Eleven of the plant extracts met this criteria with CC_50_ value less than 30 μg/mL (Table 2), with the crude methanol extract of *Macaranga barteri* and *Calliandra portoricensis* being the most cytotoxic. Incidentally, the methanol extract of *M. barteri* and *C. portoricensis* also had significant toxicity against brine shrimp nauplii. Bioactivity-guided fractionation revealed that the cytotoxic constituents of *M. barteri* lies in the dichloromethane fraction (DMB) while that of *C. portoricensis* is contained in the ethyl acetate fraction (ECP).

The primary goal of cancer chemotherapy is to specifically target cancer cells without displaying toxicity towards normal cells. This is the limitation to the use of several chemotherapeutic agents, hence, selective toxicity must be put in consideration in the discovery of leads for cancer treatment [25]. To determine whether the fractions will be cytotoxic to cancer cell lines with very minimal toxicity to normal cells, the selectivity index (SI) of the most active fraction was determined. For in vitro studies, SI value less than 1 is classified as non-selective (toxic), between 1 and 10 is weakly selective and a SI higher than 10 is considered safe (non-toxic) [26, 27]. From the selectivity studies, DMB had a SI value of 13.7 when compared to CTX with SI of 5.4 (Table 3). This means that DMB was approximately 14 times more toxic to the cancer cell line (RD) compared to normal cells (Vero), an indication that its cytotoxic activity is selective for the cancer cell line under investigation. Similarly, ECP displayed a SI value of 11.1, suggesting that this fraction, possibly possess bioactive compounds, with high selectivity for cancer cell line. However, it is essential to evaluate the cytotoxic activity of the DMB and ECP on other cancer cell lines, to have a holistic view of their activity.

*Macaranga barteri* is grown for medicinal uses and its stem is widely used as firewood in Nigeria. It is use for the management of malaria, diabetes, bronchitis and cancer in Nigeria [28, 29] and management of gonorrhoea in Sierra Leone [30]. In an ethnobotanical study conducted by our group in south west Nigeria, TMPs use the leaves of *M. barteri,* as part of herbal recipes, for the treatment of breast and skin cancer. Briefly, TMPs cook a mixture of the leaves of *M. barteri, Hoslundia opposita, Uvaria afzelii*, together with the stem bark of *Uvaria chamae, Securidaca longipedunculata,* in an earthen vessel containing clean water. Two cups (approximately 20 mL per cup) of the decoction are administered to patients three times daily for 1 month to treat the breast cancer. In addition, other traditional healers boil the leaves of *Macaranga barteri,* leaves of *Allium cepa*, leaves of *Physalis angulata* and the seeds of *Picralima nitida* in water. Two cups (approximately 20 mL per cup) of the decoction are taken twice daily for 2 months for the treatment of skin cancer.

The leaves of *M. barteri* displayed strong inhibition of lipid peroxidation in linoleic acid system and moderate reducing properties [31]. The anti-inflammatory activity of the methanol extract of the stem bark of the plant was also evaluated in a cell-based respiratory burst assay with macabarterin isolated as the compound responsible for the inhibition of superoxides in the cellular assay. Acteoside, 3,5-dicaffeoylquinic acid, kaempferol-7-*O*-glucodise and bastadin 11 were the major compounds identified in the HPLC chromatogram of DMB. Acteoside is a phenylpropanoid glycoside with anticancer, cytotoxic, anti-inflammatory and antimetastatic activities. Acteoside exhibits antiestrogenic effects on breast cancer cells and osteoblasts, without any effect on endometrial cells [32] and another study showed that acteoside inhibits human promyelocytic HL-60 leukemia cell proliferation via inducing cell cycle arrest at G0/G1 phase and differentiation into monocyte [33]. Kampferol-7-*O*-glucoside, a flavonoid glycoside, has been reported to exhibit antimicrobial and antioxidant activities [34, 35]. Ohnishi et al. reported that 3,5-dicaffeoylquinic acid showed more scavenging activities on DPPH than α-tocopherol or ascorbic acid [36]. Several bastadin analogues have been shown to possess in vitro cytotoxic activity against Sup-T1 cancer cells (T cell lymphoma) [37]. Other compounds present in small quantities in DMB include 3,6-*O*-dimethylellagic acid, viridicatol, oxaline and meleagrin. Incidentally, ellagic acid and 3-*O*-methylellagic acid have been isolated previously from the stem bark of *M. barteri* [38]. To the best of our knowledge, this is the first report on the cytotoxicity of any extract of *Macaranga barteri* on any cancer cell lines*.*

*Calliandra portoricensis* is widely used in the southwestern part of Nigeria for the treatment of prostate cancer, gonorrhoea, malaria, stomach ulcers and various gastrointestinal disorders [39, 40]. In Nigeria traditional medicine, the root of *C. portoricensis* is used, as part of herbal recipes, for the treatment of breast and prostate cancer. For the treatment of breast cancer, the grounded root of *C. portoricensis*, *Aloe vera* leaves together with the unripe fruits of *Carica papaya* and *Ananas comosus,* are soaked in pap water for 7 days. Two cups (approximately 20 mL per cup) of the infusion is taken twice daily for 1 month. To prepare the pap water, dried corn is soaked in water for 3–4 days to make it tender, after which it is blended to a paste of fine consistency. The paste is sieved to remove any corn chaff and allowed to stand for about 3 days. The liquid on top of this corn preparation (supernatant) is referred to as pap water. For the treatment of prostate cancer, the roots of *C. portoricensis*, leaves of *Andrographis paniculata*, seeds of *Lagenaria breviflora* and the roots of *Olax subscorpioides* are cooked together in an earthen vessel. A cup of the decoction is taken thrice daily for 2 months.

The aqueous extracts of both root and stem of *Calliandra portoricensis* possess anticonvulsant activity when given intraperitoneally [40] and a recent study showed that the methanol root extract of *C. portoricensis had* significant inhibitory activity on prostate cancer cell lines PC-3 and LNCaP (androgen-refractory and androgen dependent PCa-derived cell lines) growth [41]. The HPLC analysis of ECP showed that neurolenin B, nigrosporolide and 3,5,7-trimethyloctane-2,5-dienedioic were the main the major compounds. Neurolenin B has been reported as cytotoxic to human small lung carcinoma in vitro and having strong antimalarial activity against *P. falciparum*. Nigrosporolide, a 14-membered lactone with plant growth inhibitory activity inhibited the growth of etiolated wheat coleoptiles [42]. However, the chemical principle responsible for the cytotoxic activity is yet to be determined.

## Conclusion

This research revealed that eleven out of the 31 extracts investigated displayed significant cytotoxic activity, with extracts from two of the plant species (*Macaranga barteri* and *Calliandra portoricensis)*, showing cytotoxicity higher than the positive control (cyclophosphamide). This gives credence to the ethnopharmacological approach for the selection of specific plant species for natural product bio-discovery. Also, this study showed that two fractions namely: dichloromethane fraction of *Macaranga barteri* (DMB) and the ethyl acetate fraction of *Calliandra portoricensis* (ECP) displayed significant cytotoxic activity. Dicaffeoylquinic acid, acteoside, kampferol-7-*O*-glucoside and bastadin 11 identified in DMB and neurolenin B, nigrosporolide and 3,5,7-trimethyloctane-2,5-dienedioic identified in ECP, may be responsible for the observed cytotoxicity of the plant extracts. However, additional research to confirm this and/or to identify novel natural products, that in fact, are the cytotoxic compounds of interest is ongoing.

## Abbreviations

BSLA: Brine shrimp lethality assay
CTX: Cyclophosphamide
DMB: Dichloromethane fraction of *Macaranga barteri*
DMSO: Dimethylsulphoxide
DPHUI: Department of Pharmacognosy Herbarium, University of Ibadan
ECP: Ethyl acetate fraction of *Calliandra portoricensis*
FHI: Forest Herbarium Ibadan (FHI)
HPLC: High performance liquid chromatography
LD_50_: Half-maximal lethal dose
MTT: 3-(4,5-dimethyl-2-thiazolyl)-2,5-diphenyl-2-H-tetrazolium bromide, CC_50_ Half-maximal cytotoxic concentration
RD: Rhabdomyosarcoma
SI: Selective index
TMPs: Traditional medical practitioners
Vero: African green monkey kidney cells, PNT2 Human immortalised normal prostate cells
WHO: World Health Organization
